# Efficacy of Common Antimicrobial Interventions at and above Regulatory Allowable Pick-Up Levels on Pathogen Reduction

**DOI:** 10.3390/foods12040883

**Published:** 2023-02-18

**Authors:** Sabrina E. Blandon, David A. Vargas, Diego E. Casas, Oscar Sarasty, Dale R. Woerner, Alejandro Echeverry, Markus F. Miller, Carlos E. Carpio, Marcos X. Sanchez-Plata, Jerrad F. Legako

**Affiliations:** 1 International Center for Food Industry Excellence, Department of Animal and Food Science, Texas Tech University, Lubbock, TX 79409, USA; 2 Department of Agricultural and Applied Economics, Texas Tech University, Lubbock, TX 79409, USA; 3 Department of Animal and Food Science, Texas Tech University, Lubbock, TX 79409, USA

**Keywords:** ground beef, interventions, organic acid, *Salmonella*, STEC, uptake level

## Abstract

The objective of this study was to evaluate the food safety efficacy of common antimicrobial interventions at and above required uptake levels for processing aids on the reduction of Shiga-toxin producing *E. coli* (STEC) and *Salmonella* spp. through spray and dip applications. Beef trim was inoculated with specific isolates of STEC or *Salmonella* strains. Trim was intervened with peracetic or lactic acid through spray or dip application. Meat rinses were serially diluted and plated following the drop dilution method; an enumerable range of 2–30 colonies was used to report results before log transformation. The combination of all treatments exhibits an average reduction rate of 0.16 LogCFU/g for STEC and *Salmonella* spp., suggesting that for every 1% increase in uptake there is an increase of 0.16 LogCFU/g of reduction rate. There is a statistical significance in the reduction rate of Shiga-toxin producing *Escherichia coli* in relation to the uptake percentage (*p* < 0.01). The addition of explanatory variables increases the R^2^ of the regression for STEC, where all the additional explanatory variables are statistically significant for reduction (*p* < 0.01). The addition of explanatory variables increases the R^2^ of the regression for *Salmonella* spp., but only trim type is statistically significant for reduction rate (*p* < 0.01). An increase in uptake percentages showed a significant increase in reduction rate of pathogens on beef trimmings.

## 1. Introduction

Food can be contaminated during the harvest, processing, storage, distribution, transportation, and preparation phases [1]. According to the Center for Disease Control and Prevention [2], an outbreak of foodborne disease is defined as the occurrence of two or more cases of a similar illness resulting from the ingestion of a common food. A total of 841 foodborne disease outbreaks were reported in 2017, resulting in 14,481 illnesses, 827 hospitalizations, 20 deaths, and 14 food product recalls. *Salmonella* spp. accounted for 113 (29%) of the outbreaks, followed by Shiga-toxin-producing *Escherichia coli* (STEC) with 19 outbreaks (5%). Ground beef is one of the most susceptible meat products for microbial contamination and has been identified as one of the leading causes of foodborne illness, and many outbreaks have been linked to this type of product [3,4].

Ground beef has raised awareness concerning microbiological safety due to contamination from foodborne pathogens such as *Salmonella* spp. and STEC [5]. If the pathogens are present when meat trimmings are ground, then more of the meat surface is exposed to the harmful bacteria. Furthermore, grinding allows any bacteria present on the surface to be mixed throughout the meat [6]. Lymph nodes can be very difficult to remove from trimming during fabrication and oftentimes end up as part of the ground beef. This may be a potential source of pathogenic bacteria [7]. Contamination of beef trimmings increases as the product progresses through the grinding process. This can be due to increased product temperature, product homogenization, handling, and greater likelihood of exposure to surface contamination. Multiple batches of ground beef can become contaminated by a single source of contamination, but the initial bacterial load of raw materials is what ultimately determines the final bacterial population of these products [8,9].

In 2002, all raw beef processors were required by the United States Department of Agriculture-Food Safety and Inspection Service (USDA-FSIS) to undergo a reevaluation of their Hazard Analysis and Critical Control Point Systems (HACCP) plans to ensure that they were sufficiently addressing *E. coli* O157:H7 contamination [10]. Currently, USDA-FSIS collects samples of finished ground beef at inspected establishments and retail stores for the presence of *E. coli* O157:H7. Regulatory action is taken if a positive sample is collected, and the product is considered “adulterated” [11]. A majority of studies that look for pathogenic *E. coli* in raw meat have also focused not only on *E. coli* O157:H7, but also on the identification of *E. coli* serotypes such as O26, O45, O103, O111, O121, and O145. These non-O157:H7 *E. coli* serotypes are known as the “Big Six” after being listed by the United States Department of Agriculture (USDA) Food Safety and Inspection Service (FSIS) as adulterants in ground beef [12,13].

Even though *Salmonella* spp. is not an adulterant in non-intact beef, its prevalence in beef trim and ground beef is 1.27% and 4.2%, respectively [14]. In accordance with a court ruling, USDA-FSIS cannot consider *Salmonella* an adulterant of raw beef since the product must be handled properly and adequately cooked before consumption, thus destroying any pathogens [14,15]. There is evidence that the cattle hide is a major source of foodborne pathogens, including *Salmonella* spp. [16]. *Salmonella* spp. has also been found in cattle lymph nodes. During fabrication, most or all lymph nodes located in the fat tissues of beef carcasses are not necessarily removed, and it has been shown that by grinding lymph nodes with lean and fat trimmings, the lymph nodes could be a source of *Salmonella* spp. in ground beef [17].

Different intervention strategies in the form of policies, enforcement, and education are deployed to reduce risks of potential foodborne hazards. Meat processors, with the authorization of health authorities, can select, prioritize, and implement food safety interventions to reduce these risks [18]. According to the U.S. Food and Drug Administration [19], the term “antimicrobial agent” refers to a substance (including other microorganisms) or a source of radiation used to control microorganisms such as bacteria, viruses, fungi, protozoa, or other microorganisms in or on food or food contact articles. Further, in the United States, the use of validated antimicrobial interventions is an accepted principle to be able to reduce the risk of contamination during the slaughter and fabrication process. According to the FSIS [20], to consider antimicrobials as processing aids, products cannot retain more than 0.49 percent solution such that the rounded amount of water is 0 percent. The favored antimicrobial agents used in the industry are different formulations of organic acids.

Organic acids are considered food ingredients and can be produced by microorganisms. Lactic and peracetic acid are common organic acids that have been used as surface decontamination agents because they exhibit antimicrobial activities against microbiological bacteria that can be found in meat [21]. Over the past decade, peracetic acid (PAA) usage has significantly increased. There are several reasons for its adoption and acceptance, including the breakdown of PAA into acetic acid (which is the component of vinegar), water, and oxygen, as well as its acceptance by the beef and poultry industries, high stability and tolerance for organic load, and high operating efficacy at low levels [22]. Lactic acid is recognized as a natural antimicrobial agent that is safe for use in food products [23]. Using organic acid rinses in conjunction with prechill treatments on chilled carcasses before fabrication seems to provide additional safety measures by reducing the levels of pathogens [24].

Recent studies show effective reduction of pathogenic bacteria on beef surfaces with the application of organic acids by dip [25,26]. This method of application can have higher efficacy due to the increase in contact time between the intervention and the surface, but this can cause potential deterioration of sensory properties (flavor, color, and texture) [25]. The efficacy of spray application of antimicrobial agents may vary depending on the spraying system used, spraying pressure, time, and temperature. As an added benefit, the use of spray application can improve the microbiological quality of meat by reducing spoilage and pathogenic bacteria on the trimmings used for ground beef production [27,28].

Currently, the maximum threshold for uptake of antimicrobial solutions is 0.49% as a processing aid. Therefore, the objective of the study was to evaluate the optimum food safety efficacy of common antimicrobial interventions with lactic and peracetic acid at and above required uptake levels for processing aids through spray and dip applications on beef trim. As the concentration of the solutions is not considered in this threshold, only one concentration for each of the interventions was evaluated in this project (see description below in Section 2.2).

## 2. Materials and Methods

### 2.1. Preparation for Pathogen Cocktails

Seven isolates of Shiga-toxin producing *Escherichia coli* (STEC) serogroups O26 (ECRC 0.1302), O45 (ECRC 2.0164), O103 (ECRC 97.1377), O111 (ECRC 3.1009), O121 (ECRC 3.1064), O145 (ECRC 9.0538), and O157:H7 (ATCC 51657) and three *Salmonella* strains, *Salmonella* enterica subsp. enterica ser. Typhimurium (ATCC 14028), *Salmonella* enterica subsp. enterica ser. Newport (ATCC 6962), and *Salmonella* enterica subsp. enterica ser. Enteritidis (ATCC 31194) were used to inoculate and assess the efficacy of treatments.

Individual frozen isolates were transferred with an inoculation loop (1 µL) to a testing tube with 5 mL of Brain Heart Infusion Agar (BHI) (Millipore Sigma, Danvers, MA, USA) and incubated at 37 °C for 18–24 h. The isolate was then transferred from the BHI culture and streaked with an inoculation loop (1 µL) onto a plate with Tryptic Soy Agar (TSA) (Millipore Sigma, Danvers, MA, USA) and incubated 18–24 h at 37 °C. An individual colony from the plate was transferred with a sterilized cotton swab to a testing tube with 5 mL of sterilized water. The concentration of the pathogen was assessed by evaluating solution turbidity using a nephelometer (Thermo Fisher Scientific, Waltham, MA, USA) and calibrating it to 0.5 McFarland, with a McFarland standard. Turbidity of 0.5 McFarland is equivalent to 1–2 × 10^8^ CFU/mL. After confirming concentration with the nephelometer, the 5 mL of each tube was transferred to a 50 mL falcon tube containing 45 mL of Buffer Peptone Water (BPW) (Millipore Sigma, Danvers, MA, USA) to dilute the pathogen concentration by a factor of 10. Each 50 mL tube containing *Salmonella* spp. was poured into a spray bottle to use during the inoculation phase. The same procedure was followed for STEC inoculation using a separate spray bottle for inoculation. The protocol for pathogen cocktail preparation started 48 h prior to the beef trim cutting and intervention day.

### 2.2. Trim Preparation and Inoculation

Fresh trim (90/10 and 50/50; lean to fat ratio) (IMPS #138, NAMP 2014) packaged in an insulated foam shipping kit with ice packs was received each week from a beef processing plant. After arrival, beef trim was vacuum packaged by lean level and stored at 4 °C. Fresh trim was used each day of inoculation to avoid background growth of bacteria. Each trim type was cut into 20 gr (±2 gr) pieces and placed separately into a small bag (22 × 28 cm) adding up to 46 pieces per bag. Afterwards, trim was placed evenly on tray covered with aluminum foil and the cocktail (target inoculation of 10^5^ LogCFU/g) of *Salmonella* spp. or STEC was sprayed onto it (4 sprays on each tray) under a class II biosafety cabinet. After spray inoculation, trim pieces were left to sit at room temperature for 20 min for adequate attachment of bacteria. Trim weight was recorded before the intervention (lactic acid; 4.0–4.5% solution) or (peracetic acid; 390–415 ppm), which consisted of either a spray using a multi-purpose sprayer (15 psi with an acceptable spray pattern) or dip of the trim for 1, 5, and 10 s on the intervention solution, letting it sit for 1 min after immersion and then placing it back into the bag to weigh the intervened trim and measure pick up or uptake level.

### 2.3. Microbiological Enumeration

Each trim tray was split in half, where half of them went into treatment whereas the other half was enumerated immediately. After the trim had been weighed, it was transferred to a 2 L Whirl-Pak^®^ bag (Whirl-Pak, Madison, WI, USA) with filter, and 500 mL of BPW (Millipore Sigma, Danvers, MA, USA) was added. The bag was thoroughly mixed to have a homogeneous sample. Before plating the dilutions, a thin layer of 14 mL of TSA (Millipore Sigma, Danvers, MA, USA) was added to plates already containing Xylose Lysine Tergitol 4 Agar (XLT-4) (Thermo Fisher Scientific, Waltham, MA, USA) for *Salmonella* spp. and MacConkey Agar (Thermo Fisher Scientific, Waltham, MA, USA) for STEC. TSA (overlay method) was used to recover injured but viable cells affected by the interventions as validated previously by Brashears et al. [29]. Meat rinses were serially diluted and plated following the drop dilution method. Each dilution had triplicates having a total of 9 drops of 10 µL per plate. The plates were incubated for 18–24 h at 37 °C. An enumerable range of 2–30 colonies was used to report results. Bacterial counts were log transformed before statistical analysis. Attachment was evaluated, and then the enumeration after treatment was subtracted from attachment and log transformed to determine which LogReductionRate response variable was achieved per treatment combination.

### 2.4. Statistical Analysis

Linear regression analyses were performed using SAS (version 9.4) and the R software. From R, the *lm* built in function was used for estimation and the *ggplot2* package for the data visualization (version 4.1.3). Multiple models were created to assess not only the association between Uptake level on Log ReductionRate but also the effect of different organic acids, intervention methods, and lean levels as well as their interaction on the uptake levels and their effect on reducing pathogenic bacteria. The first model considered was:

(1) $$LogReductionRate=\beta_{0}+\beta_{1}Uptake+\varepsilon$$

where Log (ReductionRate) stands for reductions of STEC and *Salmonella* spp.; Uptake is percentage of retained water of beef trim; $\beta_{1}$ is a coefficient measuring the association between Uptake and LogReductionRate; and ε is the error term.

In addition to the Uptake explanatory variable, the second model also includes explanatory binary (dummy) variables for organic acid interventions (Lactic or Peracetic Acid)(Acid = 1 if peracetic acid is used, and 0 otherwise), intervention methods (Spray or Dip) (Method = 1 if spray application is used and 0 if dip method is used), and the different lean to fat ratios on the meat (90/10 or 50/50) (Trim = 1 of 90/10 lean to fat ratio is used and 0 if 50/50 lean to fat ratio is used):

(2) $$LogReductionRate=\beta_{0}+\beta_{1}Uptake+\beta_{2}Acid+\beta_{3}Method+\beta_{4}Trim+\varepsilon$$

where $\beta_{1,}$ $\beta_{2,}$ $\beta_{3,}$ and $\beta_{4}$ are coefficients related to Uptake, Acid, Method, and Trim, respectively.

The third model is an extension of Model 2 with all the possible double interactions of the binary variables.

(3) $$LogReductionRate=\beta_{0}+\beta_{1}Uptake+\beta_{2}Acid+\beta_{3}Method+\beta_{4}Trim+\beta_{5}Acid\times \mathrm{Method}\;+\beta_{6}Acid\times \mathrm{Trim}+\beta_{7}Method\times \mathrm{Trim}+\epsilon$$

where $\beta_{5}$ to $\beta_{7}$ correspond to the coefficients of the interactions between the binary explanatory variables (acid, method, and trim).

For this study, authors decided not to include bacteria as an explanatory variable because microorganisms will not affect the uptake percentage on meat; therefore, separate models were made for each pathogen.

## 3. Results and Discussion

### Reduction of Pathogens on Beef Trim

The coefficient related to Uptake ($\beta_{1}$) in the linear models represents the change in the dependent variable (Log Reduction Rate) associated with one unit increase in the uptake percentage (i.e., this is the slope of the estimated linear relation). As shown in Table 1, this coefficient is statistically significant in the three linear models. For example, as it can be observed in Model 1, the relation between STEC LogReductionRate and Uptake has a slope of 0.1646, suggesting that a 1% increase in Uptake is associated with an increase of 0.1646 LogCFU/g of reduction rate. This measure of association can be interpreted as an average estimate across all experimental conditions. As such, significant reduction rate of pathogen concentrations was achieved throughout the experiment as the uptake level increases.

**Figure 1:**
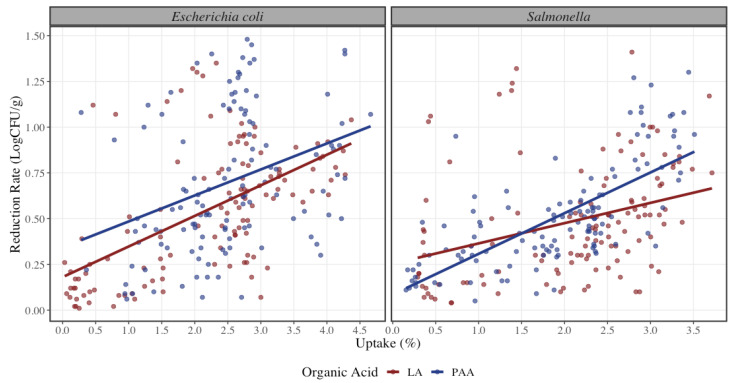
Reduction rate of Shiga-toxin producing Escherichia coli and Salmonella spp. at different uptake percentages (*n* = 240 per microorganism) with the use of lactic (red) or peracetic acid (blue). The solid line represents a linear regression model with Uptake (%) as independent variable and Reduction (LogCFU/g) as dependent variable for each pathogen bacteria. The dots represent the actual data points.

The addition of explanatory variables in Model 2 increases the R^2^ of the regression, where all the additional explanatory variables are statistically significant (*p* < 0.001) but reduce the coefficient related to Uptake. The estimated reduction rate for STEC using peracetic acid is 0.13 LogCFU/g higher than using lactic acid while keeping constant the other variables (Figure 1). Lactic and peracetic acid are common organic acids that have been used as food preservatives because they exhibit antimicrobial activities against microbiological bacteria that can be found in meat [21]. The antimicrobial activity of an organic acid can be influenced by a modification of the concentration of the antimicrobial agent or the temperature or pH at which it is acting. This modification may be considerable when it comes to their effects in terms of antisepsis, disinfection, or preservation [30].

**Figure 2:**
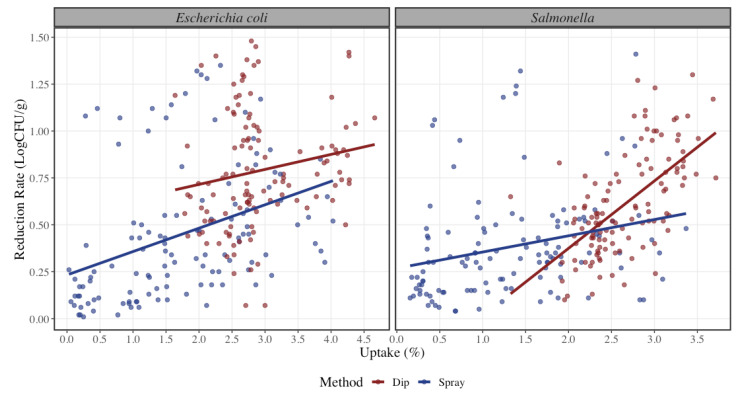
Reduction rate of Shiga-toxin producing Escherichia coli and Salmonella spp. at different uptake percentages (*n* = 240 per microorganism) by dip (red) or spray (blue) application of organic acids. The solid line represents a linear regression model with Uptake (%) as independent variable and Reduction (LogCFU/g) as dependent variable for each pathogen bacteria. The dots represent the actual data points.

Organic acids were applied by dip and spray method as shown in Figure 2. STEC reduction rate exhibits a significant difference between method of application (*p* < 0.001) where spray application results in 0.26 LogCFU/g lower reduction rate in STEC in comparison to dip application, as well as a statistical significance between the relation of uptake and method (*p* < 0.001). Similarly, trim 90/10 has a 0.28 LogCFU/g lower average reduction rate in STEC than 50/50 trim. This may be due to lack of attachment in the 50/50 trim samples making it easier to be rinsed by the organic acids. This result is similar to the study conducted by McCarty where the mostly fat covered surface had greater pathogen reduction than the mostly lean covered surface [31]. Model 3 shows that only the interaction between acid and trim is statistically significant (*p* < 0.001), which means that the difference in reduction rate of STEC between acid types depends on the trim type (and vice versa).

**Figure 3:**
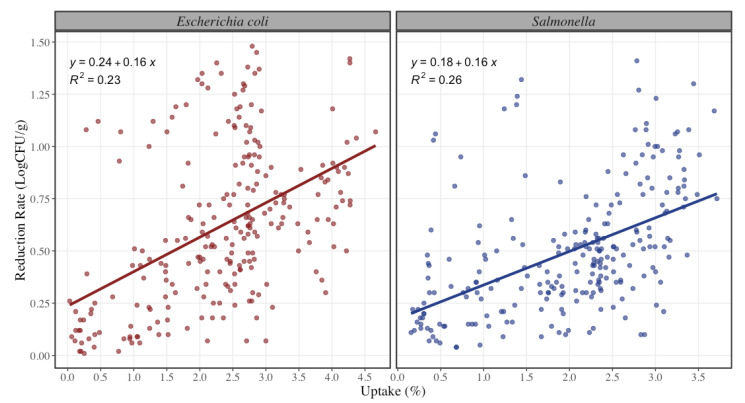
Reduction rate of Shiga-toxin producing Escherichia coli and Salmonella spp. at different uptake percentages (*n* = 240 per microorganism). The solid line represents a linear regression model with Uptake (%) as independent variable and Reduction (LogCFU/g) as dependet variable for each pathogen bacteria. The dots represent the actual data points.

The uptake percentage achieved in the study was in a range of 0.03% up to 4.66%, and the reduction rate of pathogens was from 0.2 to 1.48 LogCFU/g. In a study conducted by Signorini et al. [32], they evaluated the efficacy of decontamination of commonly used antimicrobial interventions where they demonstrated, that at higher volumes of organic acid applied, there is a greater effectiveness of the intervention, which coincides with the results obtained in this study. As shown in Figure 3, when combining all the treatments for STEC and Salmonella spp., an increase in uptake percentage is estimated to increase reduction of pathogens by 0.16 LogCFU/g or 31% (within the range of 0% to 4.6%). In a study conducted by Koohmaraie et al., organic acid reduced pathogen prevalence by 35% similar to the results shown in Figure 3 [33]. Likewise, the results obtained by Harris et al. demonstrated that antimicrobial treatments effectively reduced pathogen loads on the trim and the ground beef [34]. Other studies reported that a continued antimicrobial effect has been observed by others during storage of meat after spraying with antimicrobial solution.s and a multi-hurdle antimicrobial process can reduce the natural level of coliform bacteria in ground beef, offering an immediate reduction with a lasting inhibitory effect [24,35].

As shown in Table 2., in the three estimated models for Salmonella spp., the increase in Uptake is also found to be associated with an increase in the reduction of this pathogenic bacteria. According to Model 1, a 1% increase in Uptake is associated with a 0.16 LogCFU/g increase in the reduction rate of Salmonella spp. The addition of the binary explanatory variables in Models 2 and 3 (Table 2) increases the R^2^ of the regression, whereas the estimated coefficient related to Uptake decreases slightly.

**Figure 4:**
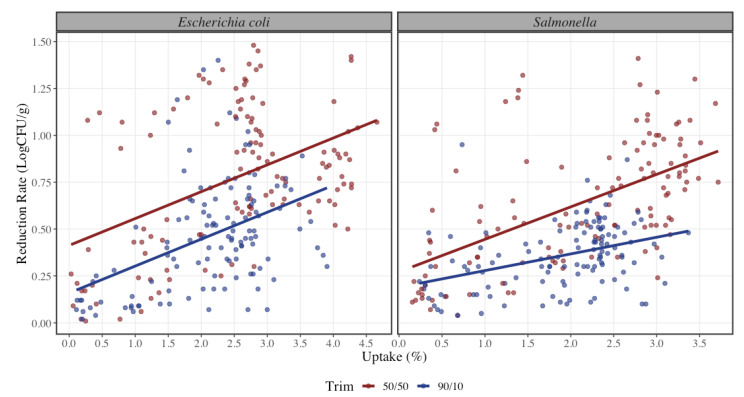
Reduction rate of Shiga-toxin producing Escherichia coli and Salmonella spp. at different uptake percentages (*n* = 240 per microorganism) when using two different lean levels (90/10 (blue) and 50/50 (red); lean to fat ratio). The solid line represents a linear regression model with Uptake (%) as independent variable and Reduction (LogCFU/g) as dependent variable for each pathogen bacteria. The dots represent the actual data points.

Regarding differences in Salmonella spp. reduction rate, there is no statistical significance between the use of lactic and peracetic acid (*p* = 0.12). Nonetheless, both organic acids achieved significant reduction rates of pathogen concentration. It was observed that at a lower uptake percentage there is a higher reduction rate with lactic acid when compared to peracetic acid, but as the uptake percentage increases, there is an increment of reduction with peracetic acid where the estimated reduction rate for Salmonella spp. using peracetic acid is 0.05 LogCFU/g higher than using lactic acid (Figure 2). This result is contrary to the findings of Ellebracht where the study suggests that peracetic acid is not as effective than lactic acid when applied by similar methods for the same amount of time [36]. For the spray application method, it was observed that it was less effective on the reduction rate by 0.06 LogCFU/g in comparison to dip application. There was no statistical significance found for the method of application in Salmonella spp. (*p* = 0.15). Overall, dip application method had an increase in reduction rate at higher uptake levels (Figure 1). These results can be compared to those of Wolf et al., where they found that reductions on dipped beef trim could have been attributed to a physical washing effect, which would not have occurred with a spray treatment [25]. During the immersion treatment, the meat may have adsorbed some of the intervention treatment solution onto the surface, thus allowing for longer exposure of the pathogen to the intervention treatment solution and providing greater pathogen inhibition. It is worth to mention that with dip application an uptake percentage lower than 1.4% could not be achieved, causing a skew of values to the right for this method of application. As the regulatory requirement is under 0.49%, a resting period would be required for draining of the excess organic acid solution. Having a resting period may also induce higher bacterial reductions, but it was not in the scope of this study. Similarly, trim 90/10 was less effective on the reduction rate by 0.25 LogCFU/g lower than 50/50 trim for Salmonella spp. Both lean levels are statistically significant (*p* < 0.001), where 90/10 trim had a lower reduction rate than 50/50 (Figure 4). Model 3 shows all the coefficients for the reduction rate of Salmonella spp. where only the interaction between peracetic acid and spray application and peracetic acid and 90/10 trim are statistically significant (*p* < 0.001).

For this study, the laboratory wanted to reproduce the same process of what we see in the industry; however, this study should be rescaled in a beef processing environment to assess efficacy of treatments in real world scenarios. Possibly with the use of pathogen surrogates to allow for measuring of LogReductionRate effectively with real size combos, treatment application, environmental variables, and resting periods.

## 4. Conclusions

Currently the USDA-FSIS requires that single-ingredient meat products cannot exceed 0.49% of retained water such that the rounded amount of water is 0%. This regulation poses a very low possibility of achieving significant reductions of pathogens on beef trimmings [20]. Thus, the objective of this study was to evaluate the food safety efficacy of common antimicrobial interventions at and above required retained water percentages for processing aids through spray and dip applications. An increase in uptake percentage of antimicrobial intervention on beef trim causes an increase in reduction rate of pathogens such as Shiga-toxin producing *E. coli* and *Salmonella* spp., thus improving the microbial safety of meat products which is now a growing concern among meat consumers. Dip application exhibits a higher reduction rate of pathogens in comparison to spray application, as well as 90/10 trim comparable to 50/50 (lean to fat ratio). Regarding organic acid, STEC showed a higher reduction rate with peracetic acid rather than lactic acid and *Salmonella* spp. had no significant difference. However, a significant 1 Log reduction is not achieved with a single application of antimicrobials.

## Figures and Tables

**Table 1:** Estimated linear regression coefficients for Shiga-toxin producing *Escherichia coli* reduction (*n* = 240) with main effects and interactions between explanatory variables.

	Model 1		Model 2		Model 3	
Variable	Coefficient (Std. Error)	*p*-Value	Coefficient (Std. Error)	*p*-Value	Coefficient (Std. Error)	*p*-Value
Intercept	0.236 (0.051)	<0.0001	0.679 (0.072)	<0.0001	0.562 (0.091)	<0.0001
Uptake	0.165 (0.020)	<0.0001	0.063 (0.021)	0.0029	0.072 (0.023)	0.0021
Acid ^a^			0.133 (0.037)	0.0004	0.289 (0.062)	<0.0001
Method ^b^			−0.265 (0.044)	<0.0001	−0.170 (0.081)	0.0363
Trim ^c^			−0.285 (0.037)	<0.0001	−0.159 (0.063)	0.0126
Acid*Method					−0.122 (0.077)	0.1136
Acid*Trim					−0.196 (0.072)	0.0071
Method*Trim					−0.476 (0.074)	0.5213
Sample size	240		240		240	
$R^{2}$	0.2254		0.4436		0.4682	
Pr > F	<0.0001		<0.0001		<0.0001	

^a^ Base organic acid is lactic acid; ^b^ Base method is dip application; ^c^ Base trim is 50/50.

**Table 2:** Estimated linear regression coefficients for *Salmonella* spp. reduction (n = 240) with main effects and interactions between explanatory variables.

	Model 1		Model 2		Model 3	
Variable	Coefficient (Std. Error)	*p*-Value	Coefficient (Std. Error)	*p*-Value	Coefficient (Std. Error)	*p*-Value
Intercept	0.146 (0.039)	<0.0001	0.381 (0.071)	<0.0001	0.408 (0.079)	<0.0001
Uptake	0.161 (0.018)	<0.0001	0.125 (0.023)	<0.0001	0.112 (0.023)	<0.0001
Acid ^a^			0.046 (0.030)	0.1206	0.089 (0.048)	0.0670
Method ^b^			−0.061 (0.042)	0.1492	0.018 (0.062)	0.7659
Trim ^c^			−0.252 (0.030)	<0.0001	−0.357 (0.052)	<0.0001
Acid*Method					−0.244 (0.056)	<0.0001
Acid*Trim					0.154 (0.056)	0.0062
Method*Trim					0.051 (0.059)	0.3847
Sample size	240		240		240	
$R^{2}$	0.2558		0.4371		0.4965	
Pr > F	<0.0001		<0.0001		<0.0001	

^a^ Base organic acid is lactic acid; ^b^ Base method is dip application; ^c^ Base trim is 50/50.

## Data Availability

Data are available on request from the corresponding author. The data are not publicly available due to privacy from the beef processing partner that allowed the project to be conducted within their beef processing environment.

## References

[1] Kamboj S., Gupta N., Bandral J.D., Gandotra G., Anjum N. (2020). Food Safety and Hygiene: A Review. Int. J. Chem. Stud..

[2] Centers for Disease Control and Prevention Surveillance for Foodborne Disease Outbreaks-United States 2017: Annual Report. https://www.cdc.gov/fdoss/pdf/2017_FoodBorneOutbreaks_508.pdf.

[3] Al-Delaimy K.S., Stiles M.E. (1975). Microbial quality and shelf-life of raw ground beef. Can. J. Public Health.

[4] Bogard A.K., Fuller C.C., Radke V., Selman C.A., Smith K.E. (2013). Ground Beef Handling and Cooking Practices in Restaurants in Eight States. J. Food Prot..

[5] Sorensen O., van Donkersgoed J., McFALL M., Manninen K., Gensler G., Ollis G. (2002). *Salmonella* spp. Shedding by Alberta Beef Cattle and the Detection of *Salmonella* spp. in Ground Beef. J. Food Prot..

[6] Food Safety and Inspection Service (2016). Ground Beef and Food Safety. https://www.fsis.usda.gov/food-safety/safe-food-handling-and-preparation/meat/ground-beef-and-food-safety.

[7] Arthur T.M., Brichta-Harhay D.M., Bosilevac J.M., Guerini M.N., Kalchayanand N., Wells J.E., Shackelford S.D., Wheeler T.L., Koohmaraie M. (2008). Prevalence and Characterization of *Salmonella* in Bovine Lymph Nodes Potentially Destined for Use in Ground Beef. J. Food Prot..

[8] Siriken B. (2004). The Microbiological Quality of Ground Beef in Aydin and Afyon Provinces, Turkey. Revue Méd. Vét..

[9] Jimenez-Villarreal J.R., Pohlman F.W., Johnson Z.B., Brown Jr A.H., Baublits R.T. (2003). The Impact of Single Antimicrobial Intervention Treatment with Cetylpyridinium Chloride, Trisodium Phosphate, Chlorine Dioxide or Lactic Acid on Ground Beef Lipid, Instrumental Color and Sensory Characteristics. Meat Sci..

[10] Arthur T.M., Bosilevac J.M., Nou X., Shackelford S.D., Wheeler T.L., Kent M.P., Jaroni D., Pauling B., Allen D.M., Koohmaraie M. (2004). *Escherichia coli* O157 Prevalence and Enumeration of Aerobic Bacteria, *Enterobacteriaceae*, and *Escherichia coli* O157 at Various Steps in Commercial Beef Processing Plants. J. Food Prot..

[11] Scanga J.A., Grona A.D., Belk K.E., Sofos J.N., Bellinger G.R., Smith G.C. (2000). Microbiological Contamination of Raw Beef Trimmings and Ground Beef. Meat Sci..

[12] Lee G.Y., Jang H.I., Hwang I.G., Rhee M.S. (2009). Prevalence and Classification of Pathogenic *Escherichia coli* Isolated from Fresh Beef, Poultry, and Pork in Korea. Int. J. Food Microbiol..

[13] Food Safety and Inspection Service (2012). Risk Profile for Pathogenic Non-O157 Shiga Toxin-Producing *Escherichia coli* (Non-O157 STEC). https://www.fsis.usda.gov/sites/default/files/media_file/2020-07/Non_O157_STEC_Risk_Profile_May2012.pdf.

[14] Bosilevac J.M., Guerini M.N., Kalchayanand N., Koohmaraie M. (2009). Prevalence and Characterization of *Salmonellae* in Commercial Ground Beef in the United States. Appl. Environ. Microbiol.

[15] Laufer A.S., Grass J., Holt K., Whichard J.M., Griffin P.M., Gould L.H. (2015). Outbreaks of *Salmonella* Infections Attributed to Beef–United States, 1973–2011. Epidemiol. Infect..

[16] Barkocy-Gallagher G.A., Arthur T.M., Rivera-Betancourt M., Nou X., Shackelford S.D., Wheeler T.L., Koohmaraie M. (2003). Seasonal Prevalence of Shiga Toxin–Producing *Escherichia coli*, Including O157: H7 and Non-O157 Serotypes, and *Salmonella* in Commercial Beef Processing Plants. J. Food Prot..

[17] Koohmaraie M., Scanga J.A., de La Zerda M.J., Koohmaraie B., Tapay L., Beskhlebnaya V., Mai T., Greeson K., Samadpour M. (2012). Tracking the Sources of *Salmonella* in Ground Beef Produced from Nonfed Cattle. J. Food Prot..

[18] Lee B. (2013). Food Safety Interventions. Natl. Collab. Cent. Environ. Health.

[19] Food and Drug Administration (2021). Microbiological Considerations for Antimicrobial Agents Used in Food Applications: Guidance for Industry. https://www.fda.gov/media/83078/download.

[20] Food Safety and Inspection Service (2002). Retained Water in Raw Meat and Poultry Products. J. Food Prot..

[21] Coban H.B. (2020). Organic Acids as Antimicrobial Food Agents: Applications and Microbial Productions. Bioprocess. Biosyst. Eng..

[22] Walsh R.J., White B., Hunker L., Leishman O., Hilgren J., Klein D. (2018). Peracetic Acid and Hydrogen Peroxide Post-Dip Decay Kinetics on Red Meat and Poultry. Food Protect. Trends.

[23] Alakomi H.-L., Skytta E., Saarela M., Mattila-Sandholm T., Latva-Kala K., Helander I.M. (2000). Lactic Acid Permeabilizes Gram-Negative Bacteria by Disrupting the Outer Membrane. Appl. Environ. Microbiol..

[24] Castillo A., Lucia L.M., Mercado I., Acuff G.R. (2001). In-Plant Evaluation of a Lactic Acid Treatment for Reduction of Bacteria on Chilled Beef Carcasses. J. Food Prot..

[25] Wolf M.J., Miller M.F., Parks A.R., Loneragan G.H., Garmyn A.J., Thompson L.D., Echeverry A., Brashears M.M. (2012). Validation Comparing the Effectiveness of a Lactic Acid Dip with a Lactic Acid Spray for Reducing *Escherichia coli* O157: H7, *Salmonella*, and Non-O157 Shiga Toxigenic *Escherichia coli* on Beef Trim and Ground Beef. J. Food Prot..

[26] ben Braïek O., Smaoui S. (2021). Chemistry, Safety, and Challenges of the Use of Organic Acids and Their Derivative Salts in Meat Preservation. J. Food Qual..

[27] Kang D.-H., Lee S.-Y. (2008). Evaluating Commercial Spray Applications of Lactic Acid, Hot Water, and Acidified Sodium Chlorite for the Reduction of *Escherichia coli* on Beef Carcasses. Food Qual. Cult..

[28] Ransom J.R., Belk K.E., Sofos J.N., Stopforth J.D., Scanga J.A., Smith G.C. (2003). Comparison of Intervention Technologies for Reducing *Escherichia coli* O157: H7 on Beef Cuts and Trimmings. Food Prot. Trends.

[29] Brashears M.M., Amezquita A., Stratton J. (2001). Validation of Methods Used To Recover *Escherichia coli* O157:H7 and *Salmonella* spp. Subjected to Stress Conditions. J. Food Prot..

[30] Russell A.D. (2008). Factors Influencing the Efficacy of Antimicrobial Agents. Principles and Practice of Disinfection, Preservation and Sterilization.

[31] McCarty K.A. (2016). Antimicrobial Interventions Applied to Beef Sub-Primals for the Control of Escherichia coli and Their Impact on Ground Beef Quality.

[32] Signorini M., Costa M., Teitelbaum D., Restovich V., Brasesco H., García D., Superno V., Petroli S., Bruzzone M., Arduini V. (2018). Evaluation of Decontamination Efficacy of Commonly Used Antimicrobial Interventions for Beef Carcasses against Shiga Toxin-Producing *Escherichia coli*. Meat Sci..

[33] Koohmaraie M., Arthur T.M., Bosilevac J.M., Guerini M., Shackelford S.D., Wheeler T.L. (2005). Post-Harvest Interventions to Reduce/Eliminate Pathogens in Beef. Meat Sci..

[34] Harris K., Miller M.F., Loneragan G.H., Brashears M.M. (2006). Validation of the Use of Organic Acids and Acidified Sodium Chlorite to Reduce *Escherichia coli* O157 and *Salmonella* Typhimurium in Beef Trim and Ground Beef in a Simulated Processing Environment. J. Food Prot..

[35] Kang D.-H., Koohmaraie M., Siragusa G.R. (2001). Application of Multiple Antimicrobial Interventions for Microbial Decontamination of Commercial Beef Trim. J. Food Prot..

[36] Ellebracht J.W. (2005). Evaluation of Peroxyacetic Acid as a Potential Pre-Grinding Treatment for Control of Enteric Pathogens on Fresh Beef Trim. Meat Sci..

